# *Campylobacter coli* of porcine origin exhibits an open pan-genome within a single clonal complex: insights from comparative genomic analysis

**DOI:** 10.3389/fcimb.2024.1449856

**Published:** 2024-10-02

**Authors:** Sandeep Ghatak, Arockiasamy Arun Prince Milton, Samir Das, Kasanchi M. Momin, Kandhan Srinivas, Daniel Aibor Pyngrope, G. Bhuvana Priya

**Affiliations:** ^1^ Division of Animal and Fisheries Sciences, ICAR Research Complex for North Eastern Hill Region, Umiam, Meghalaya, India; ^2^ College of Agriculture (CAU, Imphal), Kyrdemkulai, Meghalaya, India

**Keywords:** *Campylobacter coli*, porcine, comparative genomics, clonal complex, pangenome, pig

## Abstract

**Introduction:**

Although *Campylobacter* spp., including *Campylobacter coli*, have emerged as important zoonotic foodborne pathogens globally, the understanding of the genomic epidemiology of *C. coli* of porcine origin is limited.

**Methods:**

As pigs are an important reservoir of *C. coli*, we analyzed *C. coli* genomes that were isolated (*n* = 3) from pigs and sequenced (this study) them along with all other *C. coli* genomes for which pig intestines, pig feces, and pigs were mentioned as sources in the NCBI database up to January 6, 2023. In this paper, we report the pan-genomic features, the multi-locus sequence types, the resistome, virulome, and mobilome, and the phylogenomic analysis of these organisms that were obtained from pigs.

**Results and discussion:**

Our analysis revealed that, in addition to having an open pan-genome, majority (63%) of the typeable isolates of *C. coli* of pig origin belonged to a single clonal complex, ST-828. The resistome of these *C. coli* isolates was predominated by the genes *tetO* (53%), *blaOXA-193* (49%), and *APH (3′)-IIIa* (21%); however, the virulome analysis revealed a core set of 37 virulence genes. Analysis of the mobile genetic elements in the genomes revealed wide diversity of the plasmids and bacteriophages, while 30 transposons were common to all genomes of *C. coli* of porcine origin. Phylogenomic analysis showed two discernible clusters comprising isolates originating from Japan and another set of isolates comprising mostly copies of a type strain stored in three different culture collections.

## 1 Introduction

*Campylobacter* spp. are among the major causes of zoonotic foodborne illnesses in humans worldwide (Kaakoush et al., 2015). The available estimates have indicated that these organisms are responsible for 16% of the global foodborne illnesses (Neustaedter et al., 2023). In addition to *Campylobacter jejuni* being the well-known cause, *Campylobacter coli* is the cause of the bulk of human infections (Rosner et al., 2021; Otsuka et al., 2023). The diseases caused by these organisms range from self-limiting diarrhea, abdominal cramps, and bloody stools to other complications including Guillain–Barré and Miller–Fisher syndrome (Liu et al., 2018; Bravo et al., 2021).

*C. coli* was initially identified as *Vibrio coli*, which was believed to be the primary cause of swine dysentery for a considerably long period. The generic taxonomy underwent a change in 1963, when the name “*Campylobacter*” came into existence; thus, the organism was renamed as *Campylobacter coli* (Véron and Chatelain, 1973). The taxonomy remained largely undisputed until Vandamme and co-workers claimed that *C. coli and Campylobacter hyoilei* are subjective synonyms and that their members are indistinguishable based on their phenotypic and genotypic characteristics (Vandamme et al., 1997). Subsequently, the International Committee on Systematic Bacteriology recommended the use of *C. coli* in place of *C. hyoilei* and that the latter may be considered as a variant of the former (Vandamme and On, 2001). Since then, the status quo of the taxon has been maintained.

The infections caused by *C. jejuni* and *C. coli* are globally prevalent, with reports from many parts of the world including all of the major inhabited continents (Kaakoush et al., 2015; Sheppard and Maiden, 2015; Facciolà et al., 2017; Mourkas et al., 2020; Liu et al., 2022). The taxonomy of these gram-negative, microaerophilic, spirally curved, motile, non-spore-forming bacteria has recently been updated with the creation of a new phylum, i.e., Campylobacterota, under which 66 species are currently recognized (Kaakoush et al., 2015; Facciolà et al., 2017). In nature, *Campylobacter* spp. survive as commensal organisms in the gastrointestinal tracts of birds, livestock, and wild animals (Ayllón et al., 2017; Ocejo et al., 2023). Accordingly, these organisms have been isolated from a wide variety of sources, including poultry, milk, fruits and vegetables, water, shellfishes, pets, and flies, among many others (Facciolà et al., 2017).

Due to their substantial health burden, the pathogenicity and the virulence factors of *Campylobacter* spp., including those of *C. coli*, have been under investigation for several decades (Ishihara et al., 2006; Neal-McKinney and Konkel, 2012; O Cróinín and Backert, 2012; Rawat et al., 2018; Igwaran and Okoh, 2019). In addition to their virulence, the rising antimicrobial resistance of *Campylobacter* spp. is a matter of concern worldwide (Wieczorek and Osek, 2013; Shen et al., 2018; Elhadidy et al., 2019; Ghatak et al., 2020). Fluoroquinolone-resistant *Campylobacter* spp. have in fact been included in the global priority pathogen list as a high-priority pathogen by the World Health Organization (WHO, 2017). In the last few years, with the increased availability of whole-genome sequence data, studies analyzing the virulence and the antimicrobial resistance of *C. coli* and other *Campylobacter* spp. have increased (Ghatak et al., 2017; Gomes et al., 2020; Bravo et al., 2021; Cobo-Díaz et al., 2021; Bunduruș et al., 2023). While these reports have added to our understanding of the biology of *Campylobacter* spp., they tended to document the characteristics of only the predominant species, *C. jejuni*, with *C. coli* of porcine origin remaining underexplored. These genomic studies have also provided new insights into the evolution and the phylogenomics of these organisms (Gray and Konkel, 1999; Sheppard and Maiden, 2015). Recently, it has been highlighted that *Campylobacter* spp. possibly have evolved through a niche-segregated path adapted for various clades (Sheppard et al., 2011, 2014; Henaut-Jacobs et al., 2023). However, definitive studies focusing on the pan-genomics of *C. coli* isolated from pigs are rare. Moreover, there is a glaring lack of data on the mobilome of *C. coli*. Mobile genetic elements (i.e., transposons, plasmids, and bacteriophages) are considered important reservoirs of many virulence and antimicrobial resistance (AMR) genes that are capable of horizontal dissemination across species and genera (Partridge et al., 2018). Barring some sporadic efforts (Kim et al., 2010; Ghatak et al., 2017; Bravo et al., 2021; Quino et al., 2021), we did not come across any comprehensive analysis of these elements for *C. coli*, including those of pig origin.

Therefore, to address this deficiency in data, the present study was undertaken to comprehensively analyze the genome sequences of *C. coli* of porcine origin in order to unravel the pan-genome structure and the genomic epidemiology of this important zoonotic foodborne pathogen.

## 2 Materials and methods

### 2.1 Isolation and identification of *C. coli*

*C. coli* was isolated from retail pig offal (intestine) using the ISO 10272-1:2017 method [Institutional Animal Ethics Committee clearance no. V-11011(13)/12/2023-CPCSEA-DADF dated December 5, 2023]. Briefly, the samples were inoculated in Bolton broth (HiMedia, Mumbai, India) in a 10^−1^ dilution and were incubated for 48 h at 42°C under microaerobic condition (85% nitrogen, 10% carbon dioxide, and 5% oxygen). Following incubation, modified charcoal–cefoperazone–deoxycholate (mCCDA) agar plates were swabbed with a sterile cotton swab soaked in the inoculated Bolton broth and the inoculated plates then incubated for a further 48 h at 42°C under microaerophilic condition. Suspected colonies were examined by gram staining for colony morphology and staining characteristics. Presumptive colonies were selected and were processed for confirmation of identity.

### 2.2 Molecular confirmation of *C. coli* isolates

DNA was extracted from suspected *C. coli* isolates using the QIAmp DNA Mini Kit (Qiagen, Hilden, Germany) following the manufacturer’s instructions. For confirmation of the isolates, PCR targeting the *glyA* gene was performed as described previously (Wang et al., 2002). For further confirmation, real-time-PCR targeting the *ceuE* gene was also performed on the DNA extracted from the suspected isolates following the method described previously (Milton et al., 2020).

### 2.3 *C. coli* whole-genome sequencing and genome assembly

Genomic DNA from the *C. coli* isolates was extracted using a DNA Mini Kit (Qiagen, Valencia, CA, USA) according to the manufacturer’s instruction. Following quality assessment of the extracted DNA, whole-genome sequencing was performed on the Illumina NextSeq platform at the National Institute of Biomedical Genomics, India. Sequence data quality was assessed using the FastQC tool (https://www.bioinformatics.babraham.ac.uk/projects/fastqc/). The genomes were then assembled using the Shovill v.1.1.0 tool in tandem with the SKESA genome assembler (https://github.com/tseemann/shovill). Confirmation of the species of the assembled genomes was conducted using the Kraken2 v.2.0.7 tool (https://github.com/DerrickWood/kraken2). The final genomic sequences were deposited to NCBI (IN_20_CCNEH1_ICAR: JAQSUZ000000000, IN_20_CCNEH5_ICAR: JAQQFX000000000, and IN_20_CCNEH6_ICAR: JAQQFW000000000).

### 2.4 Additional genome data

In addition to the genomes sequenced in the present study, the genomes of *C. coli* of porcine origin (*n* = 40) (sources: pig intestines, pig feces, and pigs) were downloaded from the NCBI genome database with the cutoff date of January 6, 2023. To ensure appropriate quality control, all of these genomes were reassessed for their taxonomic affiliations using the Kraken2 v.2.0.7 tool (https://github.com/DerrickWood/kraken2). In addition to the genome quality check with the Kraken2 tool, all of the genomes included in the study were subjected to average nucleotide identity (ANI) analysis using the pyANI v.0.2.12 tool, with a 95%–96% similarity cutoff as described previously (https://github.com/widdowquinn/pyani). For ease of interpretation, the genomes were assigned codes in the format X_Y_Z, where “X” indicates the country of isolation (ISO 3166-1 Alpha-2 code), “Y” indicates the last two digits of the year of isolation, and “Z” denotes the name of the strain, if any. In the case of the same or no strain names for more than one genome, alphanumeric codes were suffixed with numbers. The genome sequences used in the study are provided in **Supplementary Table S1**.

### 2.5 Multi-locus sequence typing of *C. coli* genomes

Multi-locus sequence typing (MLST) of the *C. coli* genomes (*n* = 43) was performed with the FastMLST tool (https://github.com/EnzoAndree/FastMLST), with default settings, in combination with the PubMLST allelic profiles database for *C. jejuni*/*coli* for identification of the sequence types (STs) and the clonal complexes (CCs). The results were tabulated and analyzed further.

### 2.6 Resistome analysis of *C. coli*

The resistome of *C. coli* was determined using the Resistance Gene Identifier (RGI) v6.0.2 tool to identify the mutations conferring resistance and the resistance genes acquired by these organisms (https://github.com/arpcard/rgi). The RGI tool was run with “contig” as the input type, “Prodigal” as the ORF finder, “BLAST” as the alignment tool, and “perfect and strict,” along with the “include nudge” switch, for the identification of hits.

### 2.7 Virulome and pathogenic potential determination of *C. coli*

Virulome, the assemblage of all virulence genes of the *C. coli* genome, was determined using the ABRicate v1.0.1 tool in conjunction with the VFDB database updated up to January 19, 2023 (https://github.com/tseemann/abricate). The identification threshold for the virulence genes was set to 80% DNA coverage and 80% identity. Upon run completion, the results were summarized and further analyzed statistically. The pathogenic potentials of *C. coli* were estimated using the PathogenFinder v1.1 tool (https://cge.food.dtu.dk/services/PathogenFinder/) with “epsilon proteobacteria” as the model and “assembled genome/contigs” as the input. The results were tabulated and analyzed further.

### 2.8 Determination of the mobilome (plasmids, phages, and transposons) in the genomes of *C. coli*

All *C. coli* genomes were investigated for plasmids, phages, and transposable elements (TEs). For the plasmid sequence search, the Platon tool was utilized, with default settings (https://github.com/oschwengers/platon). Transposons within the genomes of *C. coli* were detected using the BacAnt v3.3.1 tool, in conjunction with TransposonDB v.2.0, with the default settings specified by the developer (https://github.com/xthua/bacant). Furthermore, the genomes were scanned for phage elements using the DBSCAN-SWA tool, with default settings (https://github.com/HIT-ImmunologyLab/DBSCAN-SWA). All of the results were tabulated and analyzed further for visualization.

### 2.9 Pan-genome and pan-resistome analyses of *C. coli*

Deduction of the pan-genome of the *C. coli* genomes was undertaken using the Phylogeny Enhanced Pipeline for PAN-genome (PEPPAN) v1.0.5 (https://github.com/zheminzhou/PEPPAN). To homogenize the annotation of the genomes, all of the *C. coli* genomes were annotated with Prokka v1.14.5 (https://github.com/tseemann/prokka), and the annotated files were utilized for pan-genome deduction. The pan-genome and core genome development curves were ascertained using Heap’s power law, as demonstrated previously (Tettelin et al., 2008). Furthermore, *R*_CP_, the ratio of the core genome to the pan-genome, was calculated as described previously (Ghatak et al., 2016). From the resistome data of *C. coli*, the pan-resistome and core resistome of *C. coli* were determined using the Pan-Resistome Analysis Pipeline tool (https://github.com/syyrjx-hyc/PRAP). The pan-resistome and core resistome development curves were fitted according to the power law model proposed previously (Tettelin et al., 2008).

### 2.10 Phylogenetic analysis

In order to infer the phylogenetic relatedness among the *C. coli* strains, core genome alignment was performed with Roary v.3.13.0 (https://github.com/sanger-pathogens/Roary), deployed along with the PRANK algorithm (https://ariloytynoja.github.io/prank-msa/). The obtained alignment was cleaned and stripped of poorly aligned regions using the GBlocks v0.91b tool, with default settings, for both runs (https://github.com/atmaivancevic/Gblocks). The final alignment was used as the input for the tree building tool, i.e., IQ-TREE2 (https://github.com/iqtree/iqtree2). Before construction of the phylogenetic tree, the ModelFinder tool (Kalyaanamoorthy et al., 2017) was run to identify the best model for phylogenetic reconstruction. The final tree was visualized and annotated using the FigTree v.1.4.4 tool (https://github.com/rambaut/figtree/releases). For identification of the clusters within the phylogenetic tree, the TreeLink tool (Allende et al., 2015) was used, with a cutoff value of 0.0030423472, as suggested in the algorithm.

### 2.11 Statistical analysis and data representation

Data collation and analysis were performed using MS Excel and R software (https://www.r-project.org/) implemented through R studio v.2022.02.3 Build 492. VennPainter (https://github.com/linguoliang/VennPainter) was used for the generation of Venn diagrams.

## 3 Results

### 3.1 Identification and confirmation of *C. coli* isolates

**Figure 1:**
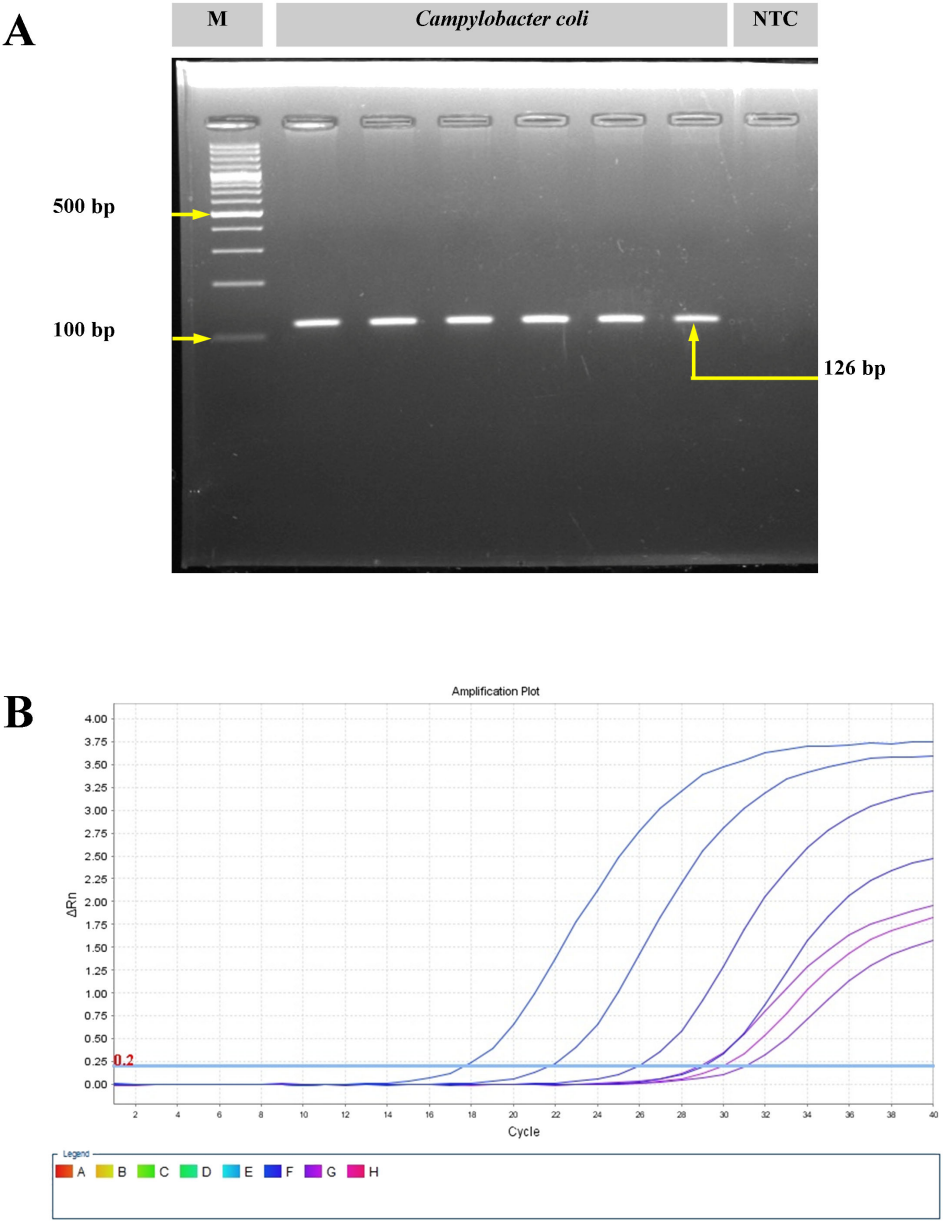
Molecular confirmation of *Campylobacter coli* with polymerase chain reaction (PCR) and real-time PCR. **(A)** Amplification of the *glyA* gene of *C*. *coli* using conventional PCR. *M*, molecular marker; *NTC*, no template control. **(B)** Amplification plot for the *ceuE* gene of *C*. *coli* with real-time PCR.

The *C. coli* isolates (*n* = 3) were isolated from pig offal (intestine) sold in retail stores in the study area (Meghalaya, India). On the mCCDA agar plates, the *C. coli* colonies were cream gray in color, were moist, and were slightly raised in appearance. All isolates yielded positive oxidase reaction. Gram-stained smears of *C. coli* revealed short, curved, rod-shaped bacteria with negative staining features. Molecular confirmation through PCR targeting the *glyA* gene yielded a 126-bp amplicon. Real-time PCR targeting the *ceuE* gene also showed positive amplification from the 18th cycle onwards (**Figure 1**).

### 3.2 Genome features

The genome dataset consisted of all the *C. coli* genomes for which pig intestines, pig feces, and pigs were mentioned as sources in the NCBI Genome database up to January 6, 2023, encompassing a total of 40 unique genomes with only one complete genome (UN_UN_RM1875; NCBI Accn. GCF_000583755.1). These genomes were obtained from four continents (Australia, Asia, Europe, and North America) and different countries, including Australia, Japan, Belgium, the Netherlands, the UK, Canada, and the USA. They were isolated from various sources/hosts, such as pig intestines, pig feces, and pigs themselves, with some sources remaining unknown.

The sizes of the genomes ranged from 1.50269 to 2.025896 Mb, with an average of 1.753125 ± 0.12 Mb. The number of coding sequences (CDS) per genome also varied, ranging between 1,539 and 2,339, with an average of 1,785 ± 160 CDS (**Supplementary Table S1**). Species check of the genomes (*n* = 43) with the Kraken2 v.2.0.7 tool (Wood et al., 2019) yielded unambiguous identification of *C. coli* for all genomes with NCBI TaxID 195. Furthermore, analysis of the ANI revealed that the minimum identity percentage among the most distant genomes under study was 98.3%, with a mean of 98.9% ± 0.003%.

### 3.3 MLST analysis of *C. coli* of porcine origin

**Figure 2:**
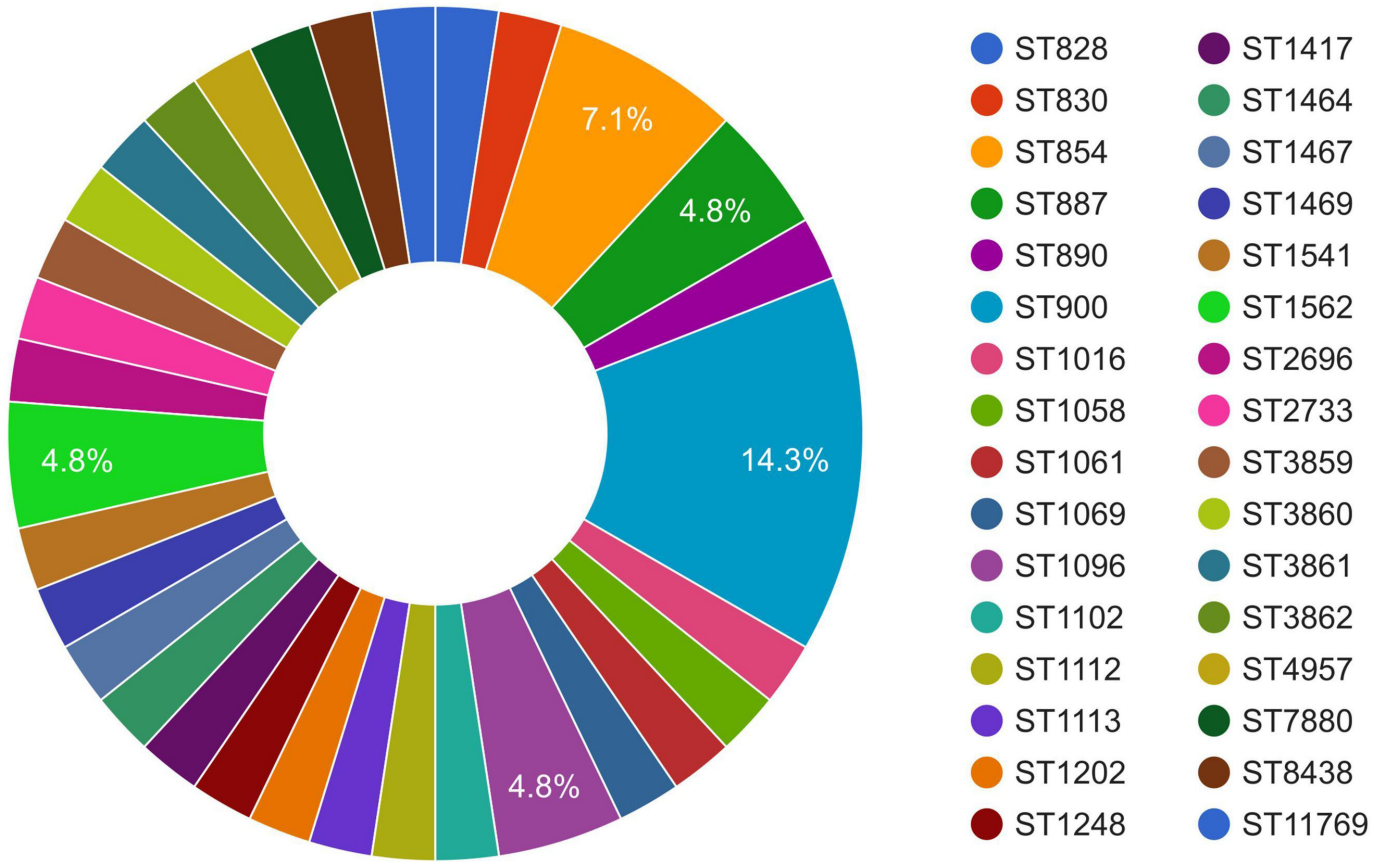
Sequence type (ST) distribution of *Campylobacter coli* of porcine origin.

MLST of the *C. coli* genomes (*n* = 43) using the FastMLST tool revealed interesting results (**Figure 2**, **Supplementary Table S1**). Of the 43 genomes of *C. coli*, 42 were typeable, while one genome (i.e., JP_13_CCP0005; NCBI BioSample #SAMD00129678) belonged to a potentially novel ST. Of the 42 typeable *C. coli* genomes, there were a wide variety of STs (*n* = 32), with ST900 being the most predominant (14.3%), followed by ST854 (7.1%). Majority of the *C. coli* genomes (27), interestingly, belonged to a single CC, i.e., ST-828.

### 3.4 Resistome of *C. coli*

**Figure 3:**
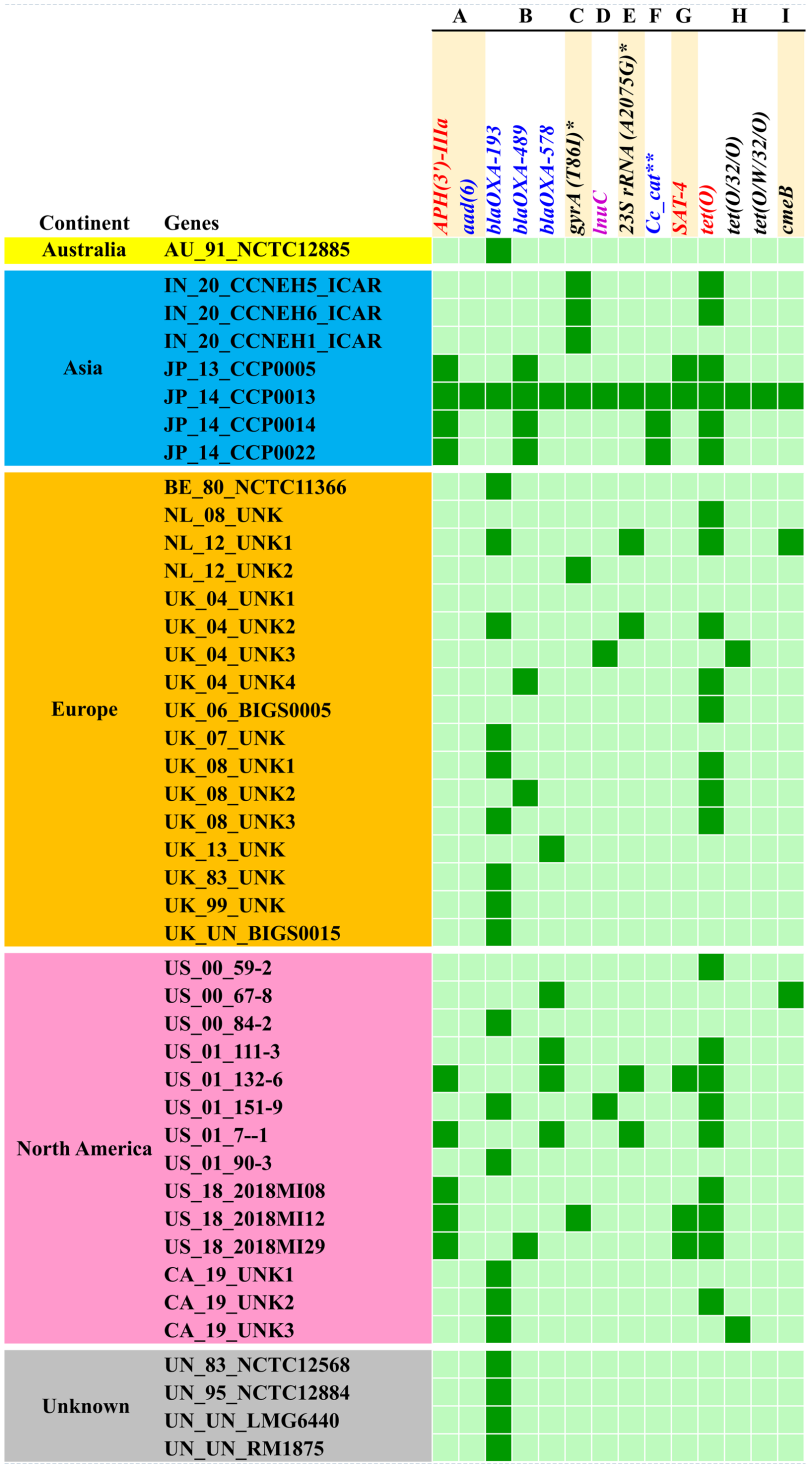
Occurrence of antimicrobial resistance genes in *Campylobacter coli* genomes. **(A)** Aminoglycosides. **(B)** Beta-lactams. **(C)** Fluoroquinolones. **(D)** Lincosamides. **(E)** Macrolides. **(F)** Phenicols. **(G)** Streptothricin. **(H)** Tetracyclines. **(I)** Multiple drug classes. Asterisk denotes mutation events. Two asterisks denote *C*. *coli* chloramphenicol acetyltransferase.

Analysis of the resistome of *C. coli* using the RGI tool with the CARD database showed occurrences of AMR genes against nine classes of antimicrobials, namely, aminoglycosides, beta-lactams, fluoroquinolones, lincosamides, macrolides, phenicols, streptothricin, and tetracyclines, as well as a group of mixed drug classes (**Figure 3**). Overall, 14 different AMR genes were observed among the *C. coli* isolates. Of these, *tetO* was the most predominant AMR gene (53% occurrence), followed by *blaOXA-193* (49%) and *APH (3′)-IIIa* (21%). When drug classes were considered, 70% of the *C. coli* genomes harbored beta-lactam resistance genes, while 58% of the genomes possessed tetracycline resistance genes and 26% of the *C. coli* genomes under study carried at least one gene imparting resistance to aminoglycoside antibiotics (**Figure 3**).

The resistome of *C. coli* showed the presence of three different genes (i.e., *blaOXA-193*, *blaOXA-489*, and *blaOXA-578*) that mediate resistance to beta-lactams; a similar number of genes (i.e., *tetO*, *tet32*, and *tetW*) were observed for tetracycline resistance. Furthermore, the resistome of *C. coli* revealed two determinants [i.e., *APH (3’)-IIIa* and *aad6*] for aminoglycoside resistance. Singleton resistance determinants against lincosamide (*lnuC*), phenicols (*C. coli* chloramphenicol acetyltransferase), and streptothricin (*SAT-4*) were also observed in the resistome of *C. coli* included in the study. In addition to the AMR genes, mutation events responsible for fluoroquinolone resistance through mutation (T86I) in the *gyrA* gene and macrolide resistance emanating from mutation (A2075G) in the *23SrRNA* gene were observed.

Breakdown of the *C. coli* genomes according to their continents of origin (Australia, Asia, Europe, and North America) indicated that the Asian isolates harbored more AMR determinants on a per genome basis. The resistomes of the *C. coli* of Indian origin (IN_20_CCNEH1_ICAR, IN_20_CCNEH5_ICAR, and IN_20_CCNEH6_ICAR) isolated and sequenced in this study revealed only a *gyrA* gene mutation (T86I) and the occurrence of the *tetO* gene.

### 3.5 Virulome analysis of *C. coli*

Analysis of the virulence genes across the genomes of *C. coli* revealed widespread occurrences of virulence genes among all isolates. Overall, 102 virulence genes were observed, which represented 14 virulence factors belonging to seven virulence classes, such as invasion, adherence, toxin, glycosylation system, secretion system, endotoxin, and chemotaxis (**Supplementary Figure S1**). Approximately 35% of these virulence genes were present in all isolates. Specifically, genes associated with the properties of invasion (*Cj0371*, *flaC*, *flaG*, *flgB*, *flgC*, *flgD*, *flgE*, *flgG*, *flgG2*, *flgH*, *flgP*, *flhB*, *flhF*, *flhG*, *fliA*, *fliE*, *fliF*, *fliG*, *fliM*, *fliN*, *fliQ*, *fliR*, *fliS*, *fliW*, *fliY*, *rpoN*, *ciaC*, and *kpsT*), adhesion (*gmhB*), flagellar glycosylation (*pseB*, *pseC*, and *pseF*), type IV secretion system (*waaF* and *waaV*), and chemotaxis (*cheA* and *cheV*) were observed in all the genomes of *C. coli*. Geographically, the average virulence gene contents per genome were 76 for Asia, 74 for Europe and North America (USA and Canada), and 85 for the genomes of unknown origin. ANOVA coupled with *post-hoc* analysis showed a significantly higher (*p* < 0.05) content of virulence genes for the last group of *C. coli*. Among the entire repertoire of virulence genes, four virulence genes (i.e., *Cj1416c* and *Cj1442c* encoding the *Campylobacter* invasion antigen, *porA* associated with the major outer membrane protein, and *cysC* conferring phytotoxin production capability) were rare and were observed only in the genomes from the USA (US_00_59-2, US_01_90-3, US_00_84-2, and US_00_59-2). Two genomes, one from Belgium, Europe (BE_80_NCTC11366), and another of unknown origin (UN_UN_RM1875), harbored the greatest number of virulence genes (87 in each genome), while the least number of virulence genes (62 in each genome) were observed in two other genomes from the USA (US_00_67-8 and US_01_7–1). On the other hand, the Indian isolates sequenced in this study possessed 70–76 virulence genes.

Assessment of the pathogenic potential of *C. coli* to cause human diseases in a scale between 0 and 1 showed that all of the *C. coli* of porcine origin were putative pathogens for humans, with probability values ranging between 0.637 and 0.816 (mean = 0.799 ± 0.008, 95% confidence interval). There was no significant difference among the *C. coli* originating from different continents (*p* > 0.05).

### 3.6 Analysis of the mobilome (plasmids, transposons, and phages) of *C. coli*

**Figure 4:**
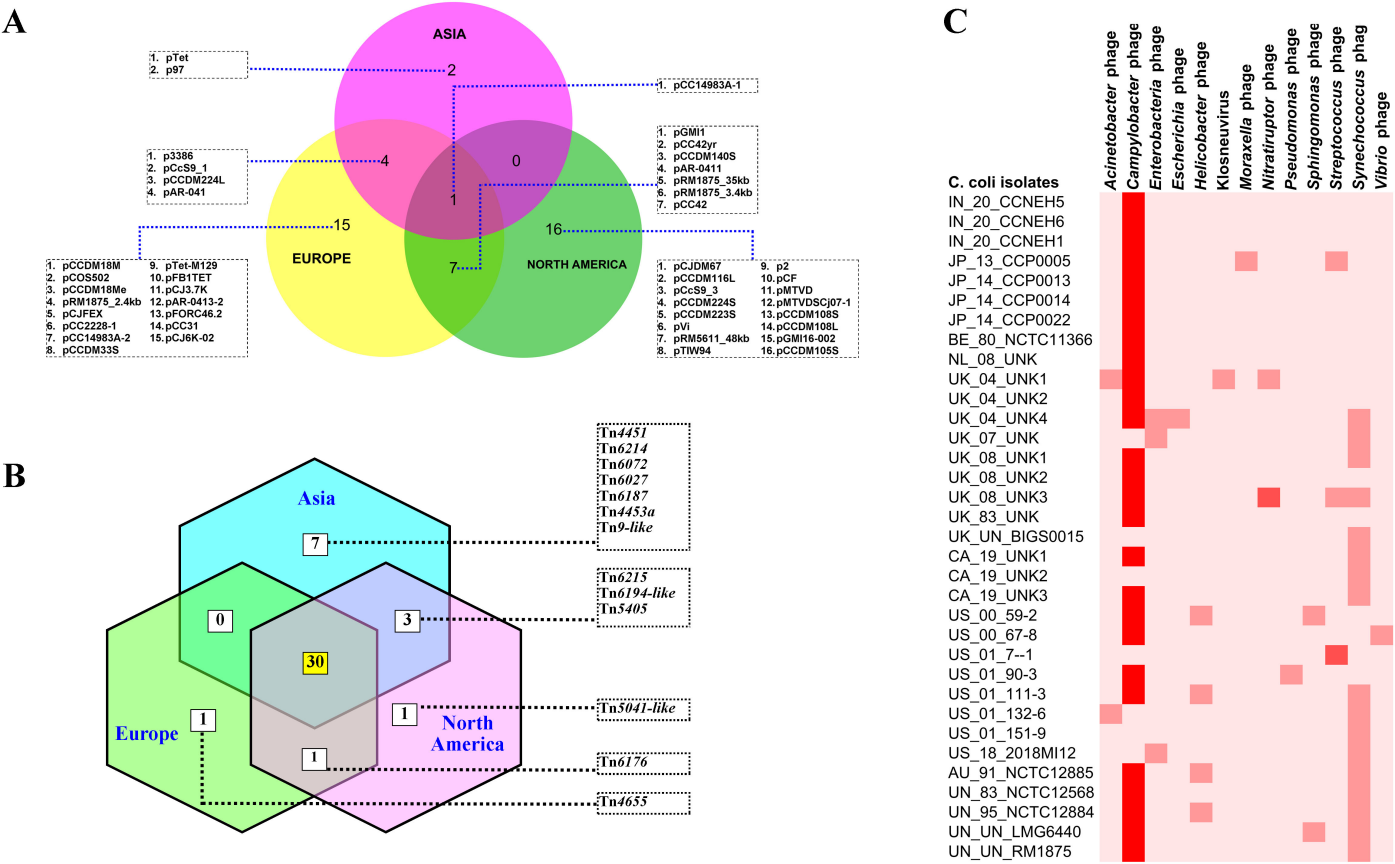
Mobile genetic elements in the genomes of *Campylobacter coli* of porcine origin. **(A)** Plasmids. **(B)** Transposable elements (TEs) (common TEs: Tn*1116*, Tn*1207.1*, Tn*1721*, Tn*1722*, Tn*1806*, Tn*2009_2*, Tn*2010*, Tn*5251*, Tn*5253*, Tn*5253-like*, Tn*5801-like*, Tn*6000*, Tn*6002*, Tn*6003*, Tn*6009*, Tn*6019*, Tn*6084*, Tn*6085*, Tn*6087*, Tn*6107*, Tn*6110*, Tn*6198*, Tn*6211*, Tn*6227*, Tn*6247*, Tn*6248*, Tn*6261*, Tn*6286*, Tn*6291*, and Tn*925*). **(C)** Bacteriophages.

To understand the features of the mobile genetic elements in the genomes of *C. coli* of porcine origin, the plasmids, the transposons, and the bacteriophages were analyzed. The results of the plasmid analysis with the Platon tool (Schwengers et al., 2020) revealed that 33 of the 43 C*. coli* genomes harbored one or more plasmids (**Supplementary Table S2**). The Platon tool identifies different plasmids by searching the marker protein sequences and the associated replicon distribution scores. In addition, the tool searches the various related genes (replication, mobilization, and conjugation), the oriT signature sequence, and the incompatibility group sequences and also performs a similarity analysis through BLAST+ against the plasmid database of NCBI. However, no statistically significant differences were observed in the numbers of plasmids carried by *C. coli* when grouped according to the continent of origin (ANOVA: *F* = 0.17961, *p* = 0.836589). However, continent-based grouping of *C. coli* showed that only one type of plasmid (pCC14983A-1) was common in all three groups (Asia, Europe, and North America) (**Figure 4A**). *C. coli* of North American and European origin possessed the largest numbers of plasmids. Interestingly, the European and the North American groups of *C. coli* also carried a large number of plasmids (15 and 16, respectively) that were unique to them. This was in contrast to the Asian isolates that possessed only two unique plasmids (pTet and p97). Overall, 33 genomes harbored a total of 45 different plasmids, indicating wide diversity in the plasmid population of *C. coli* of porcine origin.

Analysis of the transposon sequences within the genomes of *C. coli* revealed the occurrence of 43 different transposon types (**Supplementary Figure S2**). The common set of 30 transposons was present in the genomes belonging to all three continents (Asia, Europe, and North America) (**Figure 4B**). In contrast to the plasmid profiles, there were very few transposons that were unique to the genomes of the different continents. The European and North American genomes of *C. coli* had one transposon each: Tn*4655* and Tn*5041-like*, respectively. The Asian genomes of *C. coli*, on the other hand, harbored seven unique transposons: Tn*4451*, Tn*6214*, Tn*6072*, Tn*6027*, Tn*6187*, Tn*4453a*, and Tn*9-like*. The genomes of unknown and of other origins (Australia) possessed considerably fewer numbers of TEs when compared with the isolates from the other continents (**Supplementary Figure S2**).

The bacteriophages in the genomes of *C. coli* of porcine origin were determined using the DBSCAN-SWA tool, with default settings (minimum percent identity = 70%; maximum *E*-value for homology search = 1e^−7^). The results showed that 79% of the *C. coli* genomes (34 of 43) possessed signatures of a phage component belonging to 13 different virus types (**Figure 4C**). Despite the commonplace occurrences of many phage elements, the majority of the phages, for which there was a high degree of sequence similarity (71%–100%), included only *Campylobacter* phages. Other phages with moderate sequence similarity (35%–70%) included *Streptococcus* and *Nitratiruptor* phages.

### 3.7 Pangenomics

Delineation of the pan-genome structure of *C. coli* of porcine origin revealed a pan-genome comprising 3,034 genes, while the core genome of the organism consisted of 986 genes (**Supplementary Figure S3**). The analysis further revealed that the pan-genome of *C. coli* was open, with a *γ* value of 0.127, and the addition of each new genome was expected to contribute to approximately 6.8 new genes in the pan-genome. However, due to the smaller numbers of genomes, continent-based analysis was not conducted. Furthermore, the estimated *R*_CP_ of the *C. coli* genome of porcine origin was 0.325, which indicated high genomic diversity. In addition to the pan-genome description, the pan-resistome of *C. coli* was analyzed and the pan-resistome model deduced as *P*(*n*) = 2.65 *N* ^0.3^, with an *R* ^2^ value of 0.99, where *P*(*n*) is the pan-resistome and *N* is the number of genomes. The pan-resistome model also revealed an open resistome for *C. coli*, with *γ* = 0.3 (0 < *γ* < 1) (**Supplementary Figure S4**).

### 3.8 Phylogenomic analysis of *C. coli*

**Figure 5:**
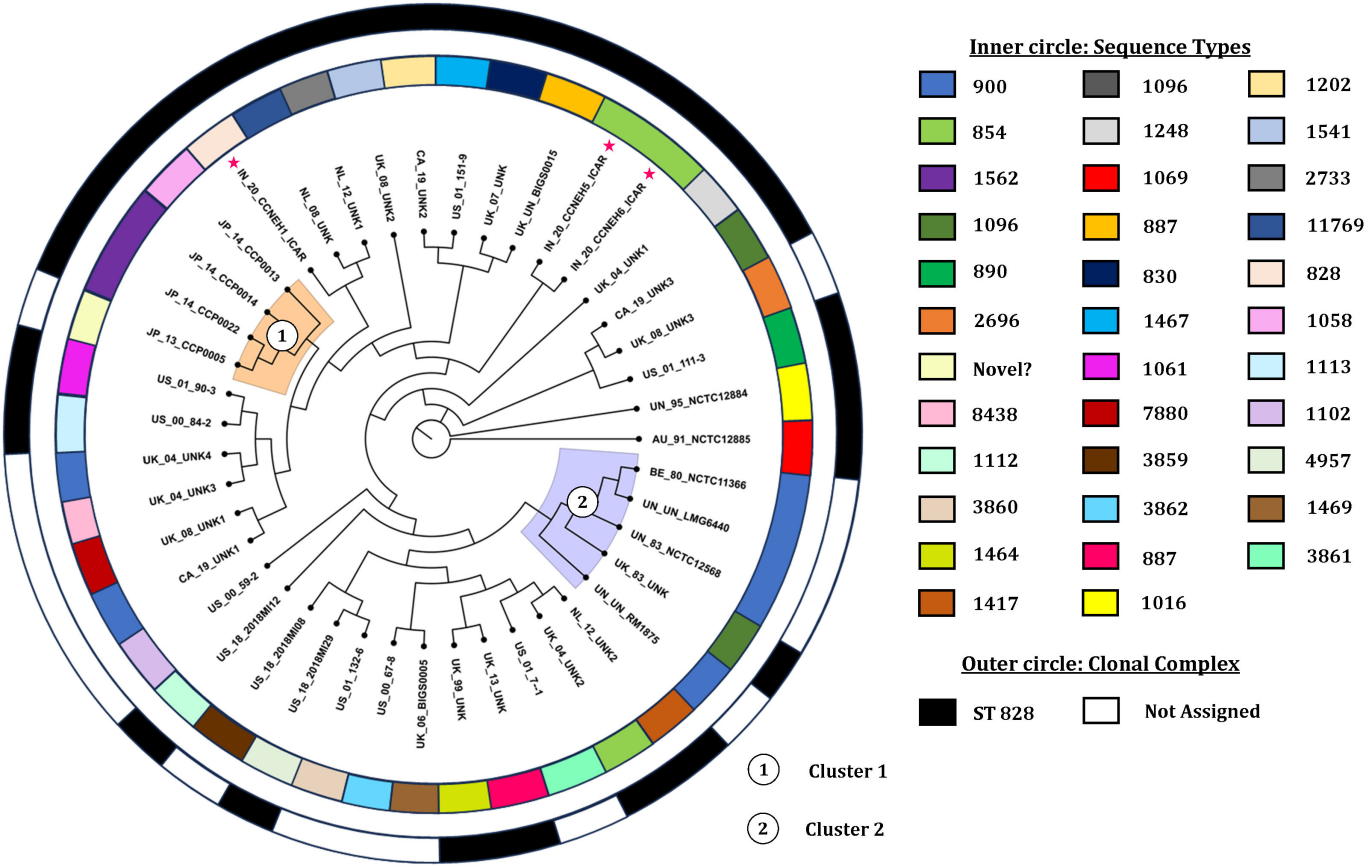
Phylogenomic tree of *Campylobacter coli* of porcine origin. Asterisk indicates the Indian isolates sequenced in this study. The genomes were assigned codes in the format X_Y_Z, where “X” indicates the country of isolation (ISO 3166-1 Alpha-2 code), “Y” indicates the last two digits of the year of isolation, and “Z” denotes the name of the strain, if any. In the case of the same or no strain names for more than one genome, alphanumeric codes were suffixed with numbers (i.e., AU, Australia; IN, India; JP, Japan; BE, Belgium; NL, Netherlands; UK, United Kingdom; CA, Canada; US, United States; UN, unknown country of origin/isolation year; UNK, unknown strain).

The core genome alignment-derived phylogenomic tree was drawn based on the best-fit model TIM2+F+I+I+R5 with the highest Bayesian information criterion score obtained from the ModelFinder run (Kalyaanamoorthy et al., 2017). The resulting tree was visualized with the FigTree v1.4.4 software (**Figure 5**). Overall, the analysis showed two discernible clusters. Cluster 1 (shaded orange) comprised the genomes originating from Japan. Cluster 2 (shaded blue) was made up of five genomes, four of which were copies of a type strain and one was of indefinite origin. There was no other definitive clustering pattern. The genomes of diverse origin were rather evenly distributed over the entire phylogenomic tree. Two of the isolates (IN_20_CCNEH5_ICAR and IN_20_CCNEH6_ICAR) were placed among a collection of *C. coli* genomes originating from the USA and the UK. The third isolate (IN_20_CCNEH1_ICAR) was more closely related to the isolates belonging to the UK and the Netherlands.

## 4 Discussion

Campylobacteriosis continues to be a leading global cause of foodborne illnesses in humans (Kaakoush et al., 2015). Among the many species of *Campylobacter*, *C. coli* is an established human pathogen (Kaakoush et al., 2015). In the natural environment, *C. coli* primarily originates from pigs and is known to be shed in quantities 10–100 times more than *C. jejuni* (Papadopoulos et al., 2021). Taking into account these compelling facts, in the present study, we reported on the genomic features of *C. coli* of swine origin from all over the world.

As part of our ongoing surveillance work on zoonotic and foodborne pathogens, we obtained three presumptive isolates of *C. coli* employing standard microbiological and molecular procedures, which were whole genome sequenced (i.e., NZ_JAQSUZ000000000.1, NZ_JAQQFX000000000.1, and NZ_JAQQFW000000000.1). To the best of our knowledge, these sequences constituted the first contributions to RefSeq of porcine origin *C. coli* from the Indian sub-continent.

MLST is a useful tool for studying the epidemiology of pathogens (Urwin and Maiden, 2003), including *C. coli* and *C. jejuni* (Elhadidy et al., 2019; Gomes et al., 2020). In our analysis, it was observed that one genome from Japan (JP_13_CCP0005) was untypeable despite passing quality checks with the Kraken2 tool and with ANI analysis (98.7% similarity), implying that this genome may well belong to a novel ST. As previously reported (Noormohamed and Fakhr, 2014; Gomes et al., 2020), we also noticed a wide diversity of STs (32 STs) among the *C. coli* genomes. However, it was interesting that two-thirds of the typeable isolates belonged to a single CC, i.e., ST-828. The predominant ST (ST900) comprised mostly copies of a type strain along with two other strains originating from Canada and the UK. Our finding confirmed that of an earlier report by Zhang et al. (2022), who reported ST900 to be the most common ST isolated from swine. On the other hand, the genomes sequenced in this study (IN_20_CCNEH5_ICAR and IN_20_CCNEH6_ICAR) and another isolate from the UK (UK_04_UNK2) made up the subset of ST854. A similar predominance of ST854 among the pig isolates of *C.* coli has been reported previously (Di Donato et al., 2020; Cobo-Díaz et al., 2021). However, the epidemiological importance of the findings could not be convincingly inferred due to the small number of genomes within each ST (ST900 and ST854). CCs are known to comprise isolates belonging to closely related STs, sharing at least four identical alleles around a “central genotype,” and are known to correspond well to common ancestry and evolutionary lineages (Sheppard and Maiden, 2015). In this context, the results of our MLST analysis provided new insights into the evolutionary epidemiological aspects of *C. coli* of porcine origin in a global scale.

Resistome analysis of the *C. coli* genomes of porcine origin using the CARD database (Alcock et al., 2020) indicated the occurrence of AMR genes against nine antimicrobial classes. The most observed AMR gene was *tetO*. A similar trend was seen in earlier studies (Cobo-Díaz et al., 2021; Hansen et al., 2021), with the predominance of the *tetO* gene among the *C. coli* genomes irrespective of the continent of origin. However, we did not find any signature of *blaOXA*-61 among the *C. coli* genomes, which is in contrast to the study by Cobo-Díaz et al. (2021). *blaOXA*-61 has been reported to be an important determinant of beta-lactam resistance in *C. coli* and *C. jejuni* (Wieczorek and Osek, 2013; Liao et al., 2022; Gomes et al., 2023). Previously, Juntunen et al. (2012) reported the occurrence of the *blaOXA*-61 gene in 77% of the *C. coli* isolated from Finnish farrowing farms. However, *blaOXA-*61-like genes include several other intrinsic oxacillinases of *Campylobacter*, which were detected in our analysis (*blaOXA*-193 and *blaOXA-*489). Moreover, the nomenclature of resistance genes continues to be debated (Lopes, 2016) and appears to be due to the evolution of *Campylobacter* oxacillinases under niche environment. Nevertheless, the absence of the *blaOXA*-61 gene was intriguing considering that *C. jejuni*/*C. coli* are noted as being intrinsically resistant to beta-lactam (Zhao et al., 2016), therefore requiring further study to understand the molecular mechanism of beta-lactam resistance in *C. coli*. Our results also revealed that *tetO*, *blaOXA*-193, and *APH (3′)-IIIa* were predominant determinants of AMR among *C. coli*. In a large-scale analysis of *C. jejuni* and *C. coli* genomes, Cobo-Díaz et al. (2021) reported a similar predominance of the *blaOXA*-193–*tet(O)*–*APH (3′)-III* gene combination among *C. coli*. Fluoroquinolone resistance in *Campylobacter* spp. is a cause of continuing concern. The results of this study indicated that the T86I mutation in the *gyrA* gene was the predominant mutation for fluoroquinolone resistance in *C. coli* from pigs. While several point mutations (e.g., A70T, D90N, T86I, and T86K) confer resistance to fluoroquinolones in *Campylobacter* spp., T86I is the most common (Shen et al., 2018). Interestingly, T86I-mediated fluoroquinolone resistance was the most common in the Asian isolates of *C. coli*, perhaps linked to the intensive use of antibiotics in the livestock sector (Bartlett et al., 2023). The results of this study also predicted open pan-resistome for *C. coli* of porcine origin. This was not unexpected, considering the global trend of increasing antibiotic use in intensive farming (Mulchandani et al., 2023) and the consequent acquisition of AMR mechanisms by bacteria (*Campylobacter* spp.) as a survival tactic (Lai et al., 2022), thereby increasing pan-resistome.

As important foodborne zoonotic pathogens, the virulence of *Campylobacter* spp. has been under discussion for many years (Ishihara et al., 2006; Rawat et al., 2018; Igwaran and Okoh, 2019). In this study, more than 100 virulence genes associated with various virulence factors were observed. Among these, a set of 37 genes encoding various virulence traits (e.g., invasion, adhesion, flagellar glycosylation, type IV secretion system, and chemotaxis) was present in all the genomes of *C. coli* of porcine origin, perhaps indicating a core virulome of *C. coli*. Statistical analysis of the occurrence of virulence genes among the four groups (three continents and one unknown) showed that the isolates of “unknown” geographical origin harbored more virulence genes. However, analysis of the predicted pathogenic potential revealed contrasting results, with no significant differences among groups. These contrasting results were perhaps indicative of the properties of the virulence genes being a more important determinant of virulence than the possession of several virulence genes by the isolates.

Plasmids play key roles in the biology of *Campylobacter*, especially with regard to antimicrobial resistance (Shen et al., 2018; Bravo et al., 2021). The results of this study revealed that the majority (76.7%) of the genomes harbored plasmids of one kind or the other, with only one common plasmid among the *C. coli*. from various continents. Our results were on the higher side compared with the previously reported plasmid, with rates of 27% and 64% for *C. coli* and *C. jejuni*, respectively (Aquino et al., 2002; Kim et al., 2010). Interestingly, of the AMR genes detected, *APH(3′)-IIIa*, *SAT-4*, and *tetO* are known to be plasmid-borne as well (https://card.mcmaster.ca/), indicating that the plasmids of *C. coli* may have important bearing on the resistome of the organism. Analysis of the TEs of *C. coli* of porcine origin revealed a contrasting picture when compared with the plasmid profile. A set of 30 TEs common to all genomes of *C. coli* from all three continents (North America, Europe, and Asia) was observed. Annotation data from the Transposon Registry database (Tansirichaiya et al., 2019) showed that many of the transposons had their origins in several genera of bacteria, including *Acinetobacter*, *Clostridium*, *Enterococcus*, *Escherichia*, *Klebsiella*, *Listeria*, *Pseudomonas*, and *Streptococcus*, possibly indicating the diversity of organisms interacting with *C. coli*. Except for a few TEs (i.e., Tn*1722*, Tn*6110*, Tn*6211*, and Tn*6291*), the major functions of TEs involve AMR. That the TEs of *C. coli* could serve as a neglected pool of AMR genes is a new insight, as discovered in this study. In this study, approximately four-fifths of the *C. coli* genomes harbored phages. The data also showed that most of the phages with high sequence similarity were *Campylobacter* phages, which was not unexpected considering the host specificity of bacteriophages. Moreover, among the phages with low similarity, a *Streptococcus* phage was detected, as well as a phage for *Nitratiruptor*, which is also a member of the phylum Campylobacterota (Ares et al., 2022).

Since the description of bacterial pan-genome and core genome, the concepts have emerged as important tools for the study of bacterial genomes, including *Campylobacter* (Bravo et al., 2021; Stoakes et al., 2023). Our analysis of *C. coli* of porcine origin revealed an open pan-genome with pan-genome and core genome sizes of 3,034 and 986 genes, respectively. The ratio between the core- and pan-genome (*R*_CP_) was considerably low (≈0.33). *R*_CP_ has previously been reported (Ghatak et al., 2016) as a tool to gauge genomic diversity, with theoretical values ranging between 0 and 1. The low *R*_CP_ value in our analysis implied that, compared with the pan-genome size, the core genome of *C. coli* was substantially smaller, indicating high genomic diversity since approximately two-thirds of the gene contents were variable. Two previous analyses comprising 4 and 12 genomes of *C. coli* reported core genome sizes of 1,294 and 1,207 (Bravo et al., 2021; Stoakes et al., 2023). Compared with these studies, the core genome size in our analysis was smaller for *C. coli* as core genome sizes are known to shrink when a larger number of genes are analyzed (Tettelin et al., 2008; Ghatak et al., 2016).

From the results of this study, we inferred the phylogenomic relatedness of *C. coli* of porcine origin reported from various countries. The results revealed two clearly identifiable clusters comprising isolates from Japan and mostly copies of a type strain for all of the *C. coli* genomes of porcine origin. The clustering patterns of the *C. coli* genomes, especially clusters 1 and 2, raised interesting possibilities. Cluster 1, comprising isolates only from Japan (an island nation), perhaps indicated a geographically constrained evolution of *C. coli* in pigs. On the other hand, cluster 2, consisting of mostly copies of a type strain, indicated that these *C. coli* genomes likely underwent very little replications between generations. Alternatively, this could also be attributed to the fact that these genomes were likely different versions of the same strain preserved in different culture collections and thus exhibited more similarity in sequences. However, it was found that the genome sequences of the type strain of *C. coli* stored in three culture collections had several differences in terms of genome size and number of coding sequences. The reasons for these variations could be attributed to sequencing errors or to minor genomic changes that occurred during sub-culturing, or both. A similar difference was observed in the case of *C. jejuni* NCTC 11168 (Gaynor et al., 2004), which opens up an avenue for future investigations.

One of the major limitations of this study is that the three genomes included in the study (BE_80_NCTC11366, UN_UN_RM1875, and UN_UN_LMG6440) might have had their origin from the same strain (Doyle, 1948). Although it could be a case of multiple representation of the same strain, we decided to be inclusive owing to the different sizes of the genomes (1.93858, 1.85872, and 1.91282 Mb) and the coding sequences (1,983, 1,877, and 1,975). This was done to avoid subjective bias on the selection of a single genome to represent a type strain. In addition, while interpreting our data, we did come across some other obstacles (such as the absence of host data, lack of phenotypic data, missing geographical data, and isolation years), which were beyond our control (for example, we did not have genomes from all continents due to unavailability in the NCBI genome database).

## 5 Conclusion

In conclusion, our analysis of *C. coli* genomes of porcine origin from all over the world unveiled interesting insights into the virulence, resistome, and mobile genetic elements, including the existence of a single clonal complex (ST-828), which should have important bearing in the control of this zoonotic foodborne pathogen.

## Data Availability

The datasets presented in this study can be found in online repositories. The names of the repository/repositories and accession number(s) can be found in the article/**Supplementary Material**.
