# Supplementary figures and images for: Campylobacter coli of porcine origin exhibits an open pan-genome within a single clonal complex: insights from comparative genomic analysis
Source: Front Cell Infect Microbiol. 2024 Oct 2;14:1449856. doi: 10.3389/fcimb.2024.1449856 (PMC11480030; doi:10.3389/fcimb.2024.1449856)

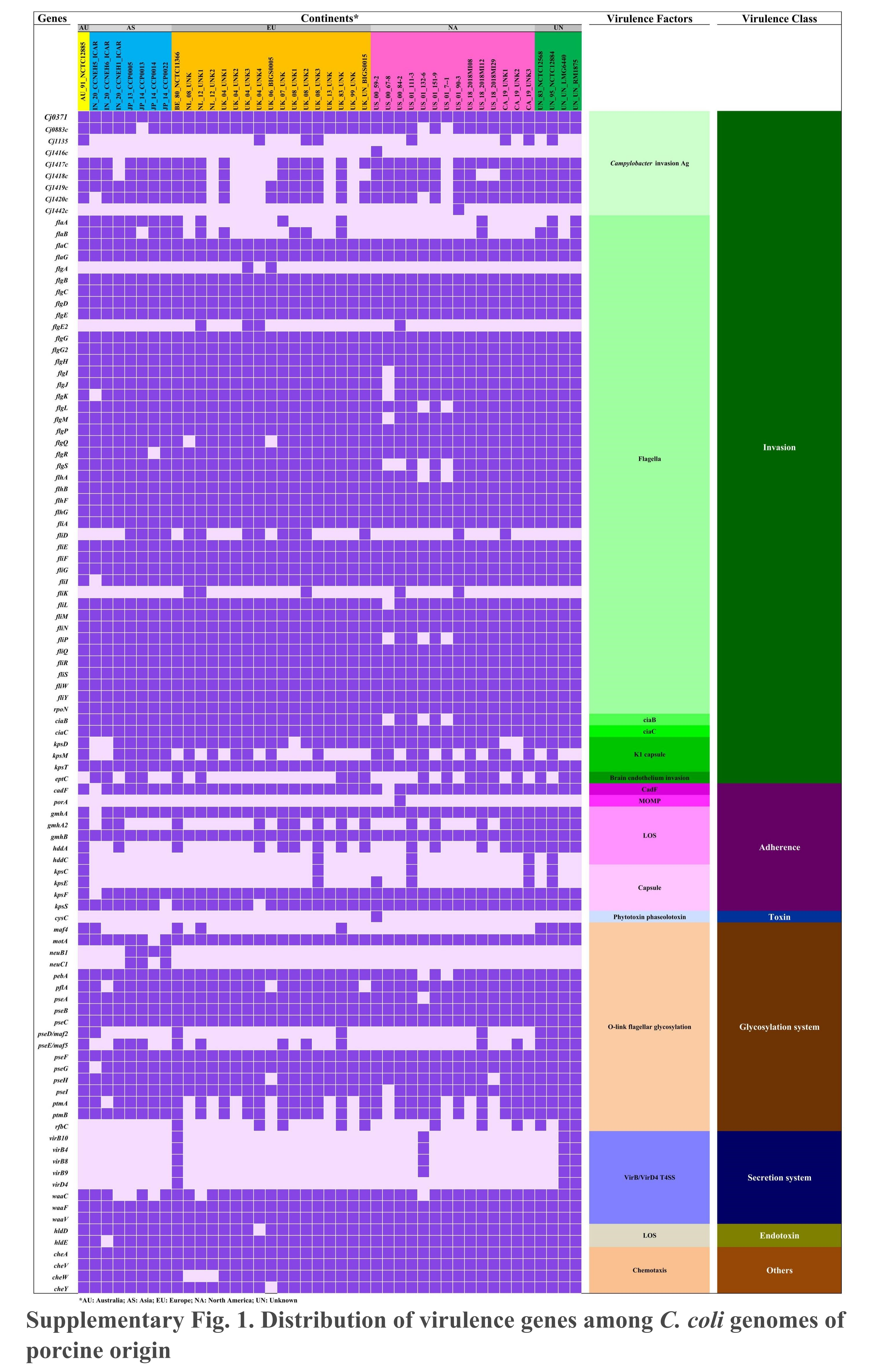

Supplement: Supplementary file 1 [file Image1.tiff]

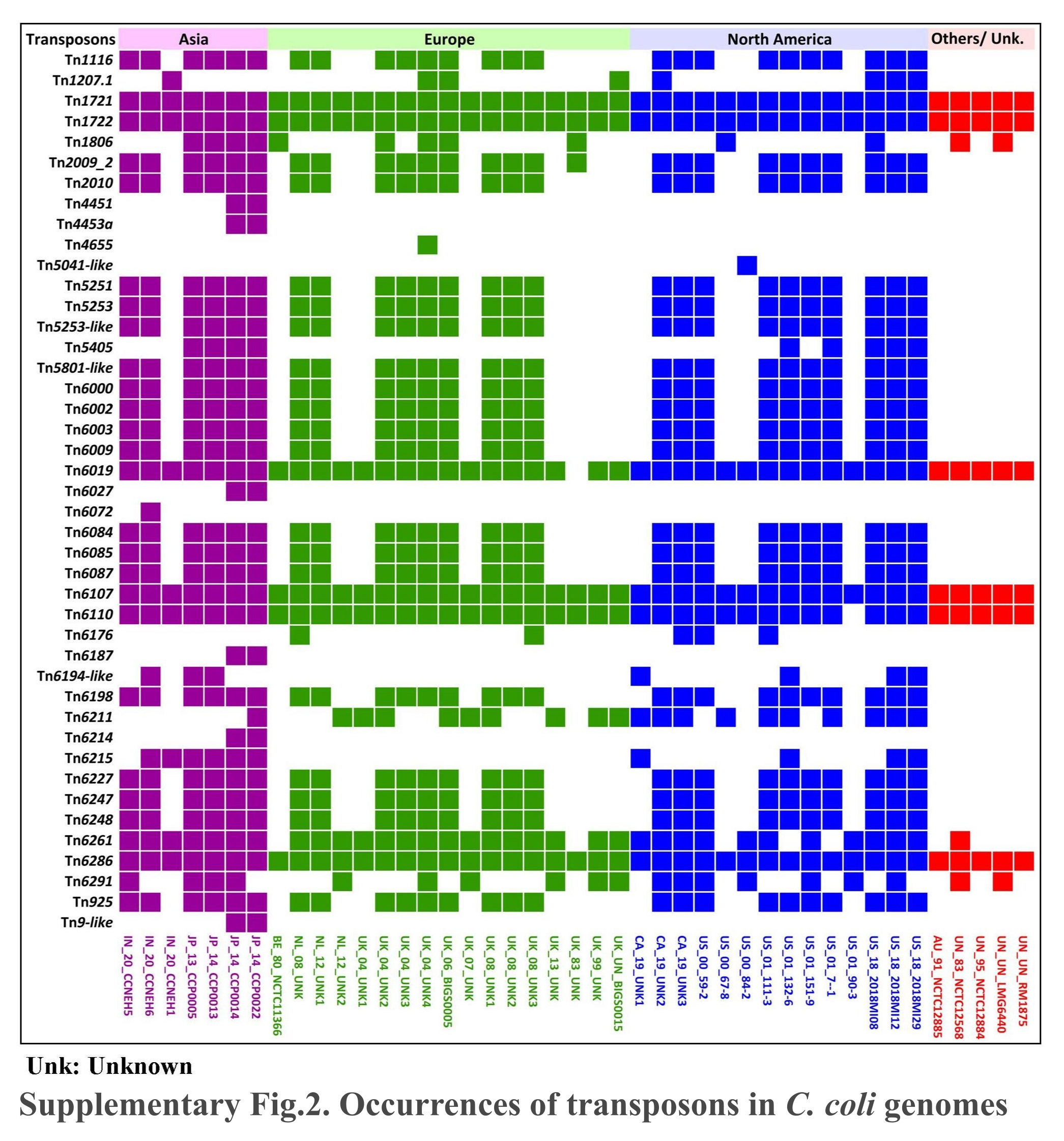

Supplement: Supplementary file 2 [file Image2.tiff]

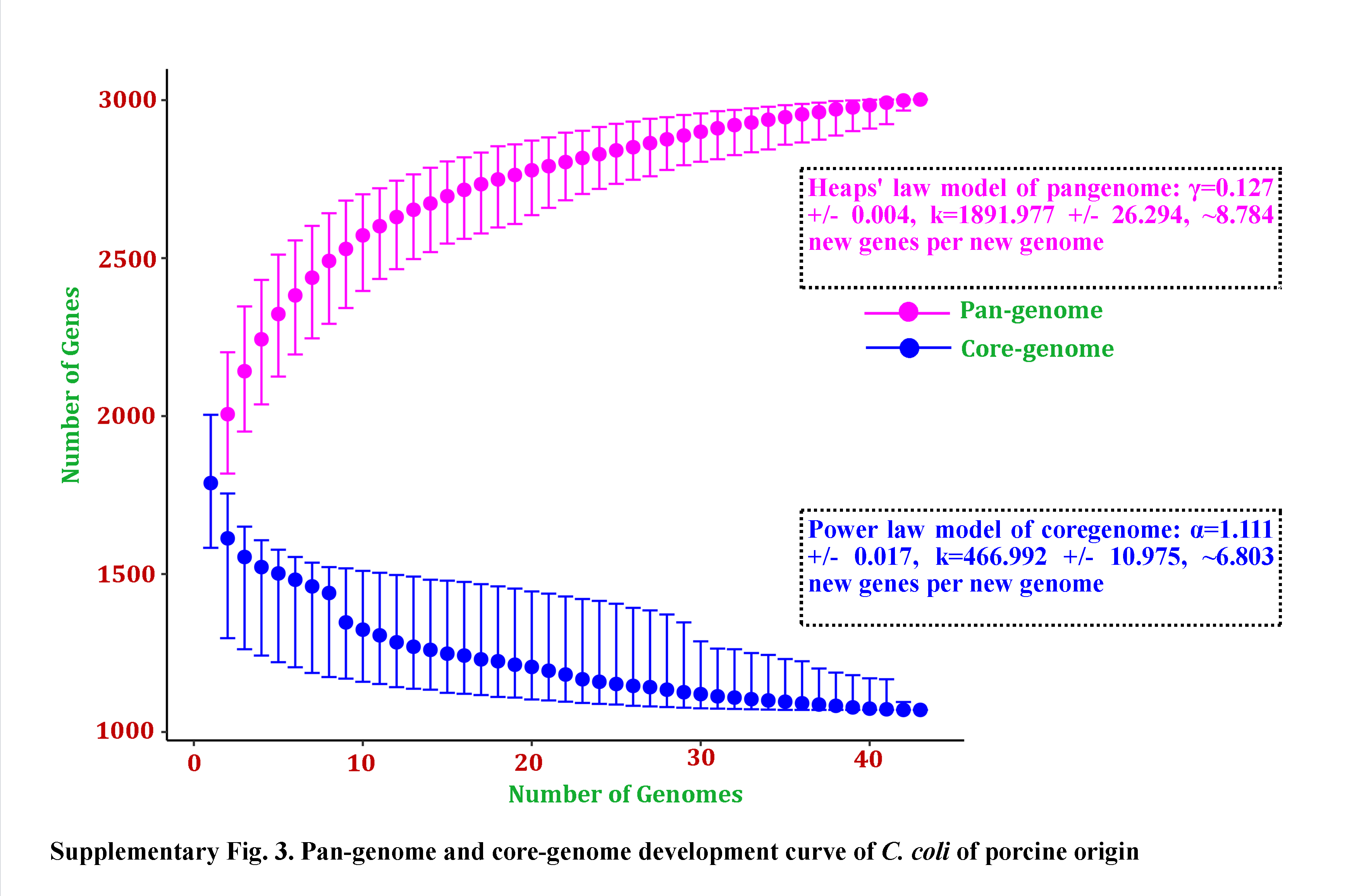

Supplement: Supplementary file 3 [file Image3.tif]

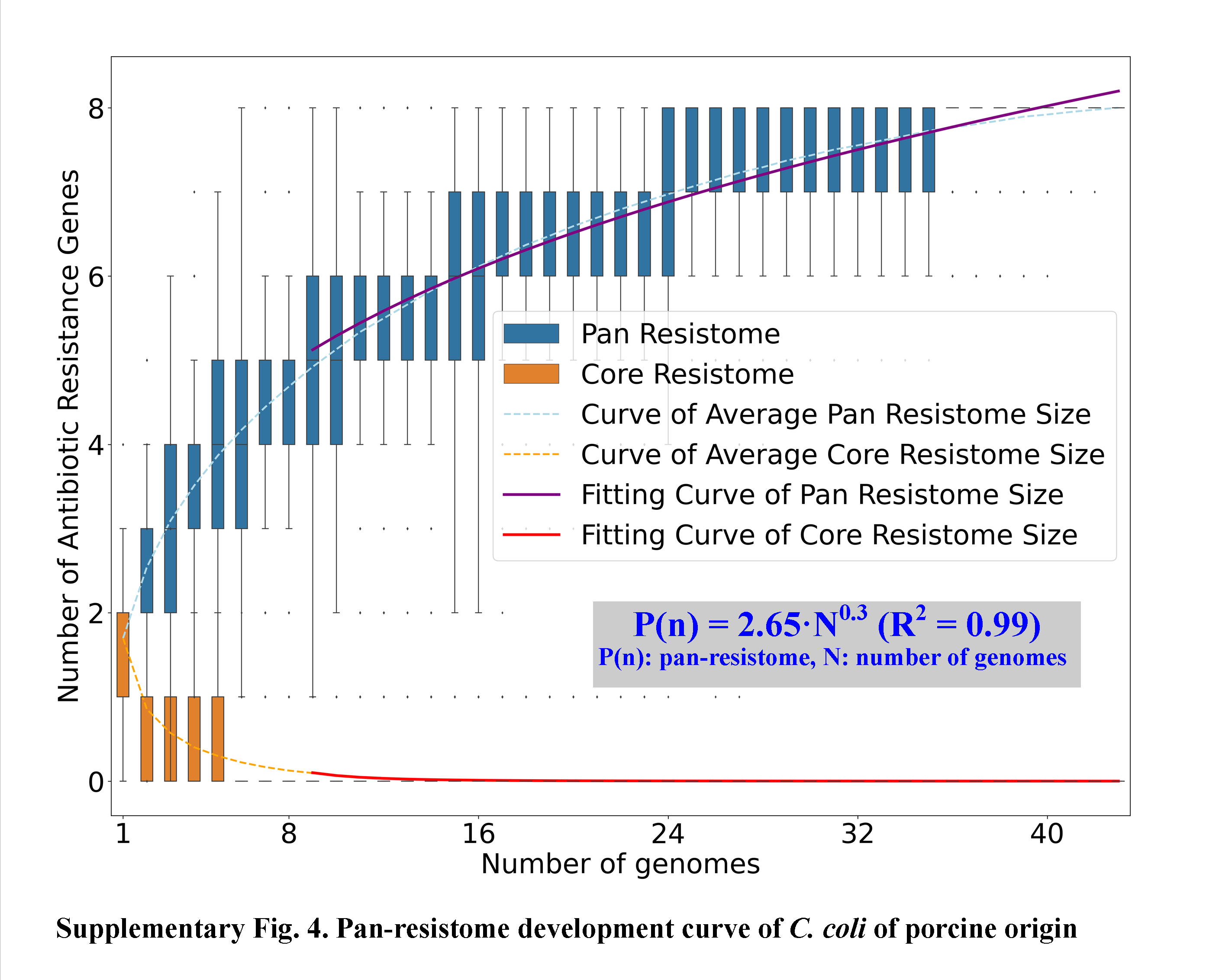

Supplement: Supplementary file 4 [file Image4.tif]
